# Guided Self-Help for Prevention of Depression and Anxiety in Women with Breast Cancer

**DOI:** 10.5402/2012/716367

**Published:** 2012-10-24

**Authors:** Hiroko Komatsu, Naoko Hayashi, Kumi Suzuki, Kaori Yagasaki, Yukiko Iioka, Joyce Neumann, Seigo Nakamura, Naoto T. Ueno

**Affiliations:** ^1^Faculty of Nursing and Medical Care, Keio University, 35 Shinanomachi, Shinjuku-ku, Tokyo 160-8582, Japan; ^2^St. Luke's College of Nursing, Tokyo 104-0044, Japan; ^3^School of Nursing, Hyogo University of Health Sciences, Hyogo 650-8530, Japan; ^4^Department of Stem Cell Transplantation and Cellular Therapy, MD Anderson Cancer Center, The University of Texas, Houston, TX 77030, USA; ^5^School of Medicine, Showa University, Tokyo 142-8555, Japan; ^6^Department of Breast Medical Oncology, MD Anderson Cancer Center, The University of Texas, Houston, TX 77030, USA

## Abstract

Depression and anxiety are prevalent in women with breast cancer. We developed a self-help kit as a self-learning package of necessary preparatory information (basic knowledge on chemotherapy, side effects, and problem-solving skills). We provided an oncology nurse-guided self-help kit with a cognitive behavioral therapy approach to 46 women with breast cancer in the intervention group and usual care to 36 in the control group in outpatient chemotherapy settings. The oncology nurse monitored and facilitated the patient's progress using the diary during the patient's chemotherapy. We also provided professional-led support group programs. Depression, anxiety, and quality of life were measured at baseline, 1 week, 3 months, and 6 months. The chi-square test and *t* were used to examine differences between the two groups, and repeated measures analysis of variance was used to test the effects of the intervention on the measures over time. All depression and anxiety scores were improved in both the intervention and control groups, but there were no significant differences between the two groups. Further studies are needed to evaluate the effectiveness of an oncology nurse-guided self-help approach for cancer patients.

## 1. Introduction

It is well established that nonpharmacological management using cognitive behavioral therapy (CBT) is effective for the management of depression and anxiety [1–3], and for maintaining quality of life (QOL) in cancer patients [4]. Given the limited availability of therapists, the access is a challenge [3]. Low-intensity working with less practitioner time may contribute to a wider use of CBT [1]. “Guided self-help” defined as a self-administered intervention facilitated by healthcare professionals using a range of books or evidence-based self-help manuals for specific purposes, was recently recommended in the National Institute for Health and Clinical Excellence (NICE) guidelines for the management of depression in adults with a chronic physical health problem [5].

Psychological need is one of the highest unmet supportive needs of cancer patients, and increases with longer time from diagnosis [6]. Surviving cancer usually means enduring sequential combinations of treatment modalities, including surgery, radiotherapy, chemotherapy, and hormonal treatment. It is stressful for most women to confront not only the burden of treatment, but also the fear of recurrence and death. The prevalence of depression, anxiety, or both is two-fold higher in women with early breast cancer than in the general female population [7].

Women with breast cancer need support to cope with the psychological consequences of the disease [8], but many feel insufficiently informed about psychological support options [9]. Despite the recognition that early preventive intervention against psychological distress soon after cancer diagnosis is critical, there are few management strategies [10]. 

Through our development of people-centered care to promote patient self-help and to establish the best possible care system as part of the 21st Century Center of Excellence program [11], we found that women with breast cancer often faced psychological challenges during the treatment process [12]. Chemotherapy has a significant impact on QOL of women with breast cancer [13], and may increase the risk of depression and anxiety [7, 14]. Professional-led support groups for cancer patients have been shown to have positive effects on the psychosocial functioning of their members [15]. Encouragement and support are needed to achieve self-management [16]. Oncology nurses are the most valuable clinical contact, and can provide the initial education and psychological support to cancer patients [17–19].

In this study, we investigated the effectiveness of the oncology nurse-guided self-help intervention compared with usual care in preventing the development of depression or anxiety in women with breast cancer in addition to support group programs in outpatient chemotherapy settings.

## 2. Materials and Methods

### 2.1. Participants and Settings

This study was approved by the Institutional Review Board of St. Luke's College of Nursing and by the Institutional Review Board of St. Luke's International Hospital. Patients were invited to join the study at an outpatient appointment before their chemotherapy commenced at an ambulatory treatment center in a general hospital in Tokyo, Japan. We obtained written informed consent from all participants before commencing the study. We included women diagnosed with primary breast cancer who were younger than 80 years of age, had been surgically treated and were awaiting adjuvant therapy (4 cycles of epirubicine combined with cyclophosphamide: EC therapy). Participants were excluded if they had relapsed, were pregnant, or had a history of mental disorder. We assigned patients receiving EC therapy for the first year of the study to the control group and those receiving therapy for the second year to the intervention group. 

### 2.2. Intervention

The intervention group received a 12-week guided self-help intervention (Table 1). We developed a self-help kit as a self-learning package of necessary preparatory information during a period of outpatient chemotherapy. The self-help kit aimed at helping patients develop cognitive representations based on rehearsing the outpatient chemotherapy procedure; improving patients' belief in their ability to manage side effects; and helping patients build problem-solving skills. 

An orientation was given by the nurse, and all participants saw the video on chemotherapy procedure before the initial adjuvant chemotherapy. The participants received self-learning reading materials (basic knowledge on chemotherapy, side effects, and problem-solving skills), and kept diaries for self-management. To reinforce participant learning, the nurse in attendance monitored and facilitated the patient's progress using the diary during the patient's chemotherapy, which was administered every 3 weeks. 

We also provided professional-led support groups for patients to attend at least two to three times during the study period to promote effective management of psychological and emotional responses through interaction with peers in support groups. A small support group meeting comprising about five members was held once a week in the communication lounge next to the treatment room on outpatient visit day. On the basis of a prepared scenario, two topics were discussed in the support group (for example, “How do you talk about your cancer to the people close to you?” or “How do you balance your treatment and daily living?”). Each support group meeting was facilitated by one of the investigators and the nurse from an ambulatory treatment center and lasted 70 minutes. 

Women assigned to the control group received usual care from the nurse using a simple chemotherapy education leaflet which was different from that for the intervention group. The leaflet included information on adverse events and management, and emergency contact (e.g., in case of fever). We completed all outcome measures within the same timeframe as that of the intervention group. 

### 2.3. Nurse Training

To assure the quality of the intervention, the 13 nurses who provided interventions attended a 2-day training session consisting of two workshops. The purpose of the first session was to teach the nurses the theory and practical method of the guided self-help program. To assess the nurses' level of understanding of the instruction method, we conducted pre- and posttests using a computer-assisted learning. The nurses answered 18 true or false questions on the knowledge and information necessary to improve coping processes in daily living and with side effects during chemotherapy. Posttest scores were significantly higher (*P* < .01) than pretest scores, and the knowledge base of the nurses who participated in the session was deemed sufficient, as we reported elsehwere [20]. The purpose of the second session was to teach the nurses how to facilitate the support group. The nurses were divided into small groups of 2 or 3, and each nurse roleplayed discussion on how to cope with treatment and living. 

### 2.4. Measures

#### 2.4.1. Center for Epidemiologic Studies-Depression Scale

Patients' reports of depression symptoms were evaluated by the Center for Epidemiologic Studies-Depression (CES-D) Scale, a 20-item self-report questionnaire to measure depression symptoms in four domains (depression affect, somatic complaints/activity inhibition, positive affect, and interpersonal difficulties) on a four-point scale during the week prior to evaluation [21]. Higher scores on this measure indicate higher levels of depressive symptomatology, with scores equal to or greater than 16 indicating an increased risk of clinical depression. We used the Japanese version of the CES-D [22], which is a common tool to for psychological assessment of patients with breast cancer in Japan [23].

#### 2.4.2. Spielberger State-Trait Anxiety Inventory

The Spielberger State-Trait Anxiety Inventory (STAI) contains two 20-item forms used to measure state anxiety (the level of present anxiety) and trait anxiety (the general level of anxiety experienced) [24]. Higher scores indicate greater anxiety. We used the Japanese version of the STAI scale [25], since it is widely used to assess psychological distress of patients with breast cancer in Japan [26]. 

#### 2.4.3. Short Form-36 Health Survey

The Short Form-36 (SF-36) Health Survey is a generic questionnaire used to measure two major health concepts (physical and mental health) with 36 questions and 8 multiitem scales: physical functioning, role functioning physical, bodily pain, general health, vitality, social functioning, role functioning emotional, and mental health [27]. Higher scores indicate better health. We used the Japanese version of the SF-36 because of a reliable self-reporting tool for patient populations in Japan [28]. 

Toxicity was graded according to adverse events of chemotherapy assessed by administering the Japanese version of NCI-Common Toxicity Criteria*：* NCI-CTC v2.0 [29]. 

Data were collected at baseline (T1), 1 week (T2), 3 months (T3), and 6 months (T4). The data at baseline and 1 week were collected on site, while the participants returned completed questionnaires at 3 months and 6 months by mail. The study period was from November 2002 to March 2006. 

### 2.5. Statistical Analysis

We used the chi-square test and the Student *t*-test to examine differences between the two groups. We performed repeated measures analysis of variance (ANOVA) to test the effects of the intervention on the measures over time. All statistical analyses were performed using two-tailed tests, and the significance level was set at 0.05. 

## 3. Results

### 3.1. Patient Characteristics

**Figure 1:**
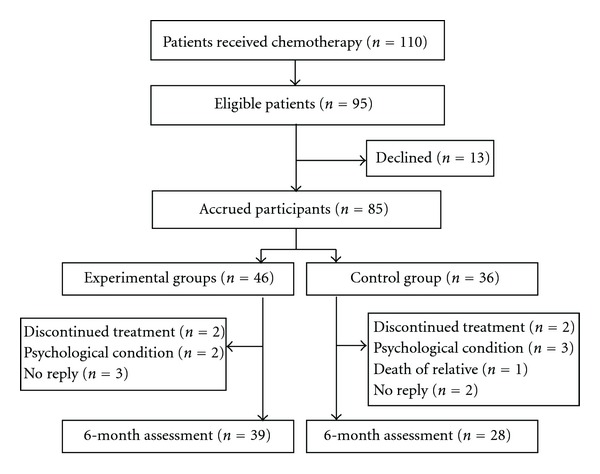
Flow chart of eligible patients for the study.

Of the 110 female patients who received postoperative adjuvant systemic therapy, 95 met the eligibility criteria for recruitment. Thirteen patients refused to participate; thus, a total of 82 patients (46 in the intervention group and 36 in the control group) consented to participate. At the time that T4 questionnaires were completed (6 months postinvestigation), 7 patients (15.2%) had withdrawn from the intervention group, leaving a total of 39, and 8 (22.2%) had withdrawn from the control group, leaving 28. The reasons for withdrawal in the intervention group included discontinued treatment (*n* = 2), psychological condition potentially hampering compliance with the study protocol (*n* = 2), and no reply (*n* = 3). Similarly, in the control group, discontinued treatment (*n* = 2), psychological condition potentially hampering compliance with the study protocol (*n* = 3), loss of a family member (*n* = 1), and no reply (*n* = 2) were the reasons for withdrawal (Figure 1).

There were no significant differences in patient demographic or medical characteristics (age, employment, education, stage of cancer, and treatment) at baseline between the intervention and control groups (Table 2). Adverse events included mucositis, fatigue, emesis, nausea, and alopecia, and there were no significant differences in toxicity between the two groups. 

### 3.2. Effect of the Intervention on Anxiety and Depressive Symptoms

Table 3 shows the mean scores for anxiety and depression symptoms at T1, T2, T3, and T4 in the intervention and control groups. At baseline, there were no significant differences between the intervention and control groups on the trait anxiety or state anxiety scales, and the repeated measures ANOVA revealed no significant differences in the state anxiety or trait anxiety scales between the two groups over the study period. Although the depression levels on the scales decreased markedly in the intervention group over time, none of these bivariate relationships were significant. The effect size ranged from 0.000 to 0.001.

### 3.3. Effects of the Intervention on Quality of Life

At baseline, there were no significant differences between the intervention and control groups on any SF-36 subscales (Table 4). The effect size ranged from 0.000 to 0.024. Although only the mental subscale scores showed a significant difference between the two groups over the study period, the effect size was small (*F* = 7.48,  *P* = .008,  *η* ^2^ = 0.004). 

## 4. Discussion

In this study, the guided self-help intervention was not significantly more effective than the usual care, although all depression and anxiety measures and QOL measures were improved in both the intervention and control groups.

### 4.1. Possible Explanations

There are possible explanations for the present results, including sample size, the severity of disease, placebo effect, and number of contact. 

Patients who experience clinically significant depression, may be more likely to be benefit from the intervention. Badger et al. [30] report that women with a high depression burden had the greatest gains in psychological adjustment with five 6-week self-help interventions, and Pitceathly et al. [31] also demonstrated that a 3-session (face-to-face and telephone) program by nonspecialists was an effective intervention for cancer patients at high risk of developing anxiety or depressive disorder. Therefore, in a population that does not have a high risk of depression but does have a primary diagnosis of cancer, a larger sample size or a longer followup may be needed to detect significant differences in the STAI and CES-D. 

Repeated contact with the same nurse might have provided those in the control group with a feeling of security [7], even though they received no intervention but only routine chemotherapy sessions with the standard explanations. 

The contact number of the nurse was six in the intervention group, which was within the range of no less than three contacts and no more than six recommended by the NICE guideline [5]. The intensity of intervention, however, might not have been sufficient. Although the effects of follow-up telephone calls for cancer patients were demonstrated in other studies [31, 32], the nurse talked to the patient only during the injection of anticancer drug at the ambulatory treatment center in this study due to the limited staffing. 

Furthermore, the instruments we used in the present study are common for measuring changes in depression and anxiety, and QOL. These instruments, however, are not designed specifically for use in cancer patients, and this may have undermined the results of this study.

### 4.2. Future Research

Depression and anxiety are common in cancer patients. As the role of oncology nurses is on the rise, how the oncology nurse can contribute to prevention and management of depression and anxiety remains a challenge. Efforts are reported across continents. In Canada, there is a movement to implement a standardized screening tool for distress by oncology nurses on a routine basis [33]. A randomized nursing intervention using a stepped care approach, in which the basic concept is similar to this study, is planned in Sweden [34]. Assessment and intervention for depression and anxiety in cancer patients by oncology nurses, guided self-management in particular, needs further research.

### 4.3. Limitations

The findings of the present study need to be interpreted in the context of several limitations. We did not randomly assign individual patients to groups in order to avoid a possible influence that could be caused by two or more patients visiting the same outpatient clinic on the same day. Since the intervention group data was collected after the control group data, there exists the possibility of threatening external validity due to changes over time. Our recruitment rate was lower than initially planned and resulted in the relatively small sample size, because we recruited patients by strict inclusion criteria of chemotherapy regimen (EC therapy only). And finally, the nurses' intervention in the patient's self-help was limited to chemotherapy sessions, and the intensity of intervention therefore might not have been sufficient.

## 5. Conclusions

The present study found that the guided self-help intervention for depression and anxiety was not significantly more effective the usual care, although all depression and anxiety measures and QOL measures were improved in both groups. 

## Figures and Tables

**Table 1:** Guided self-help intervention.

	Contents
Self-help kit	Orientation on chemotherapy by nurse at outpatient clinic.
	Video on chemotherapy procedure.
	Self-learning reading materials:
	(1) Basic knowledge on chemotherapy.
	(2) Management of side effects.
	(3) Problem-solving skills.
	“Life during chemotherapy” using diary for self-management.
	Discussion with the nurse about the patient's progress.

Support group	70-minute weekly meeting facilitated by nurse with coping scenarios
	(1) Mood control.
	(2) Coping with treatment and daily living.

**Table 2:** Demographic and medical characteristics of the participants (*n* = 67).

	Intervention	Control	
Demographic and medical variables	*n* = 39	*n* = 28	*P*
	*n* (%)	*n* (%)	
Patient demographics			

Mean age in years ± S.D.	47.7 ± 8.0	49.5 ± 10.9	NS
Employment status			
Working part-time or full-time	12 (30.8)	6 (21.4)	
Housewife	15 (38.4)	13 (46.4)	NS
Sick leave/retired	12 (30.8)	9 (32.2)	
Education			
<Bachelor's degree	22 (56.4)	18 (64.3)	NS
>Bachelor's degree	17 (43.6)	10 (35.7)	
Stage of breast cancer			
I	7 (18.0)	6 (21.4)	
II	27 (69.2)	21 (75.0)	
III	5 (12.8)	1 (3.6)	
Type of surgery			
Breast-conserving	26 (66.7)	20 (71*．*4)	
Mastectomy	13 (33.3)	8 (28.0)	

**Table 3:** STAI and CES-D scores by intervention and control groups.

	T1		T2		T3		T4				Effect size	
	Mean (SD)	95% CI	Mean (SD)	95% CI	Mean (SD)	95% CI	Mean (SD)	95% CI	*F*	95% CI	*P*	*η* ^2^
STAI (state) (20–80)												
Intervention (*n* = 39)	52.5 (11.3)	48.87–56.21	44.8 (11.7)	40.98–48.56	41.1 (10.6)	37.62–44.49	38.4 (11.2)	34.75–42.02	2.95	41.29–47.09	0.091	0.001
Control (*n* = 28)	49.3 (11.2)	44.98–53.66	43.8 (10.6)	39.66–47.85	42.7 (9.9)	38.86–46.57	39.3 (9.2)	35.77–42.87		40.35–47.20		

STAI (trait) (20–80)												
Intervention (*n* = 39)	45.7 (11.5)	42.01–49.48	43.8 (11.0)	40.20–47.34	41.4 (12.1)	37.48–45.34	40.1 (10.7)	36.60–43.51	2.83	39.82–45.67	0.097	0.000
Control (*n* = 28)	42.9 (11.4)	38.47–47.31	42.9 (9.8)	39.04–46.68	43.3 (9.1)	39.73–46.77	40.9 (9.6)	37.17–44.62		39.03–45.92		

CES-D (0–60)												
Intervention (*n* = 39)	17.0 (11.4)	13.30–20.70	13.5 (11.3)	9.80–17.12	12.1 (10.5)	8.66–15.44	10.0 (8.1)	7.40–12.61	0.71	10.64–15.62	0.53	0.000
Control (*n* = 28)	15.1 (11.5)	10.66–19.56	14.4 (8.2)	11.24–17.62	13.1 (9.9)	9.31–16.98	9.3 (6.9)	6.67–12.04		10.07–15.95		

**Table 4:** SF-36 scores by intervention and control groups.

		Time 1			Time 2			Time 3			Time 4						Effect size
	*n*	Mean	SD	95% CI	Mean	SD	95% CI	Mean	SD	95% CI	Mean	SD	95% CI	*F*	95% CI	*P* value	*η* ^2^
Physical functioning: PF																	
Intervention	39	85.9	12.8	81.74–90.05	87.7	12.2	83.74–91.64	89.1	9.5	86.04–92.17	87.7	19.1	81.52–93.87	0.09	84.86–90.34	0.766	0.024
Control	28	84.6	10.4	80.59–88.69	81.8	9.7	78.01–85.56	86.4	8.9	82.97–89.88	86.8	13.9	81.4–92.20		81.68–88.14		

Role-physical: RP																	
Intervention	39	34	35.1	22.59–45.36	37.2	40.5	24.05–50.31	55.8	42.7	41.92–69.62	72.4	31.3	62.29–82.58	0.12	41.57–58.11	0.943	0.010
Control	28	25	31.2	12.91–37.09	33	35.4	19.31–46.76	53.6	40.1	38.03–69.12	67	40.9	51.12–82.81		34.88–54.40		

Bodily pain: BP																	
Intervention	39	52.8	21.8	45.76–59.88	66.3	19.3	60.03–72.53	66.3	20.2	59.72–72.84	70.3	20.6	63.66–77.01	0.48	59.42–68.44	0.493	0.000
Control	28	53.9	20	46.11–61.60	68	16.6	61.58–74.42	65.8	21.9	57.34–74.30	67.7	18.9	60.36–75.00		58.52–69.16		

General health: GH																	
Intervention	39	51.3	16.5	45.95–56.62	52	16.5	46.61–57.34	55.6	16.8	50.18–61.10	58.6	18.1	52.73–64.45	1.25	50.10–58.64	0.268	0.006
Control	28	56.1	15.1	50.30–61.99	53	13.1	47.96–58.11	58.8	16	52.53–64.97	58	15.1	52.13–63.87		51.44–61.52		

Vitality: VT																	
Intervention	39	53.8	20.3	47.26–60.43	54.5	22.9	47.06–61.91	61.7	22.2	54.46–68.87	67.4	21.8	60.36–74.51	3.11	53.80–64.92	0.082	0.010
Control	28	54.8	20.4	46.92–62.73	53.9	15.4	47.97–59.88	55.4	23.5	46.23–64.48	59.1	21.7	50.68–67.53		49.24–62.36		

Social functional: SF																	
Intervention	39	57.1	27.3	48.19–65.91	57.7	27.9	48.65–66.74	67.6	25.4	59.38–75.88	78.8	21.1	72.01–85.69	0.15	59.54–71.07	0.699	0.001
Control	28	59.8	22.1	51.24–68.41	54	24.1	44.69–63.35	66.1	23	57.14–75.01	77.7	23.4	68.60–86.76		57.60–71.20		

Role-emotional: RE																	
Intervention	39	43.6	42	29.98–57.21	53	47	37.77–68.22	69.2	41.5	55.79–82.67	76.1	31.5	65.87–86.28	0.21	51.29–69.66	0.647	0.015
Control	28	36.9	42.9	20.28–53.54	38.1	41.3	22.08–54.11	65.5	44	48.43–82.52	72.6	41.6	56.48–88.76		42.44–64.12		

Mental health: MH																	
Intervention	39	53	21	46.23–59.82	64.2	23	56.75–71.67	71.5	21.1	64.66–78.32	76.6	17.4	70.98–82.26	7.48	61.45–71.22	0.008*	0.004
Control	28	60.1	20.8	52.06–68.22	64.4	18.6	57.20–71.66	63	21.2	54.78–71.23	69.9	14.7	64.17–75.54		58.59–70.13		

*P < .05.
